# Mowat-Wilson syndrome

**DOI:** 10.1186/1750-1172-2-42

**Published:** 2007-10-24

**Authors:** Livia Garavelli, Paola Cerruti Mainardi

**Affiliations:** 1 Clinical Genetics Unit, Obstetric and Pediatric Department, S. Maria Nuova Hospital, Reggio Emilia, Italy; 2 Department of Pediatrics and Clinical Genetics, S. Andrea Hospital, Vercelli, Italy

## Abstract

Mowat-Wilson syndrome (MWS) is a multiple congenital anomaly syndrome characterized by a distinct facial phenotype (high forehead, frontal bossing, large eyebrows, medially flaring and sparse in the middle part, hypertelorism, deep set but large eyes, large and uplifted ear lobes, with a central depression, saddle nose with prominent rounded nasal tip, prominent columella, open mouth, with M-shaped upper lip, frequent smiling, and a prominent but narrow and triangular pointed chin), moderate-to-severe intellectual deficiency, epilepsy and variable congenital malformations including Hirschsprung disease (HSCR), genitourinary anomalies (in particular hypospadias in males), congenital heart defects, agenesis of the corpus callosum and eye anomalies. The prevalence of MWS is currently unknown, but 171 patients have been reported so far. It seems probable that MWS is under-diagnosed, particularly in patients without HSCR. MWS is caused by heterozygous mutations or deletions in the Zinc finger E-box-binding homeobox 2 gene, *ZEB2*, previously called *ZFHX1B *(SIP1). To date, over 100 deletions/mutations have been reported in patients with a typical phenotype; they are frequently whole gene deletions or truncating mutations, suggesting that haploinsufficiency is the main pathological mechanism. Studies of genotype-phenotype analysis show that facial gestalt and delayed psychomotor development are constant clinical features, while the frequent and severe congenital malformations are variable. In a small number of patients, unusual mutations can lead to an atypical phenotype. The facial phenotype is particularly important for the initial clinical diagnosis and provides the hallmark warranting *ZEB2 *mutational analysis, even in the absence of HSCR. The majority of MWS cases reported so far were sporadic, therefore the recurrence risk is low. Nevertheless, rare cases of sibling recurrence have been observed. Congenital malformations and seizures require precocious clinical investigation with intervention of several specialists (including neonatologists and pediatricians). Psychomotor development is delayed in all patients, therefore rehabilitation (physical therapy, psychomotor and speech therapy) should be started as soon as possible.

## Disease name

Mowat-Wilson syndrome

## Definition

Mowat-Wilson syndrome (MWS; MIM# 235730) is a genetic disease caused by heterozygous mutations or deletions of the *ZEB2 *gene, and characterized by typical face, moderate-to-severe mental retardation, epilepsy and variable congenital malformations, including Hirschsprung disease (HSCR), genital anomalies (particularly hypospadias in males), congenital heart disease (CHD), agenesis of the corpus callosum (ACC) and eye defects. The clinical aspects of the syndrome were first described by Mowat *et al *in 1998, who also identified a locus at chromosome 2q21-q23 [1]. In 2001, two groups independently discovered the cause of MWS as mutation or deletion of the *ZEB2 *gene (MIM# 605802) from studies of two *de novo *translocations and demonstrated intragenic truncating mutations in several other individuals affected by the disease [2,3].

## Epidemiology

The prevalence of MWS is currently unknown, but it seems probable that the syndrome is under-diagnosed, particularly in patients without HSCR [4]. Since the first delineation by Mowat *et al *(1998), approximately 171 patients with *ZEB2 *mutations, deletions or cytogenetic abnormalities have been reported primarily from Northern Europe, Australia, Italy and the United States, and over 100 mutations have been described [2-31]. The male/female ratio is approximately 1,42:1 [19,22,24,29-31]. The syndrome has been identified in several ethnic groups [22], with similar clinical features in all populations.

## Clinical description

Clinical manifestations of the MWS are presented in Table 1.

**Table 1:** Mowat-Wilson Syndrome: Clinical features in patients with *ZEB2 *mutations [1-31].

**Clinical features**	**Published cases (n = 171)**
Facial gestalt	166/170 (97%)
M:F	100:70
Mental retardation	all, moderate, usually severe
Microcephaly	135/166 (81%)
Seizures	102/139 (73%)
HSCR	97/170 (57%)
Constipation	19/73 (26%)
CHD	87/167 (52%)
Urogenital/renal anomalies	81/156 (51%)
Hypospadias	33/63 (52%)
Cryptorchidism	23/63 (36%)
Renal anomalies	20/156 (12.8%)
Short stature	34/73 (46%)
Hypoplasia or agenesis of corpus callosum	67/155 (43%)
Pyloric stenosis	8/170 (4.7%)
Structural eye anomalies	7/170 (4.1%)
Cleft palate	5/170 (2,9%)
Pulmonary artery sling with/without tracheal stenosis/hypoplasia	5/167 (2.9%)
*ZEB2 *mutations	All

### Facial gestalt

**Figure 1:**
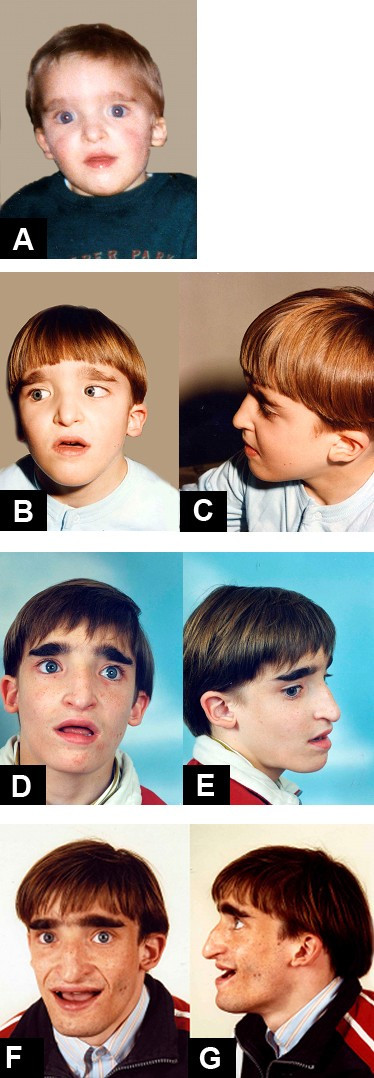
Mowat-Wilson Syndrome, clinical features of *Patient 1 *at age: (A) 1 year and 6 months; (B-C) 5 years; (D-E) 13 years and 8 months; (F-G) 18 years.

**Figure 2:**
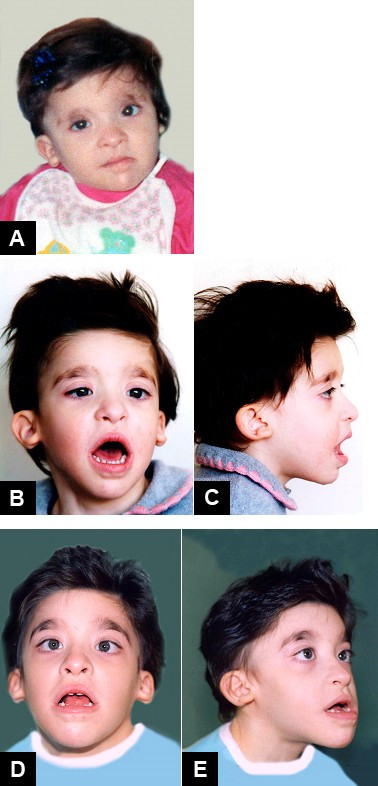
Mowat-Wilson Syndrome, clinical features of *Patient 2 *at age: (A) 1 year and 6 months; (B-C) 3 years and 5 months; (D-E) 8 years and 1 month.

**Figure 3:**
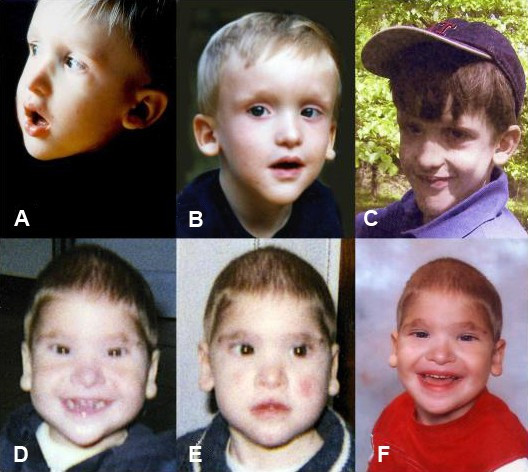
Mowat-Wilson Syndrome, clinical features of *Patients 3 and 4 *at age: *Patients 3: *(A) 1 year and 2 months; (B) 3 years and 4 months; (C) 8 years and 1 month; *Patients 4: *(D) 3 years; (E) 3 years; (F) 3 years and 6 months.

The clinical features of the face are very typical and the diagnosis of MWS is based on recognition of the distinctive facial gestalt, usually associated with severe mental retardation. This phenotype, which changes with age, typically prompts the clinician to consider the diagnosis (Figures 1, 2, 3).

**Figure 4:**
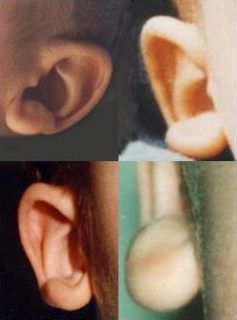
Main common features of the patients with Mowat-Wilson Syndrome: uplifted ear lobes as "orecchiette pasta" or "red blood corpuscles".

**Figure 5:**
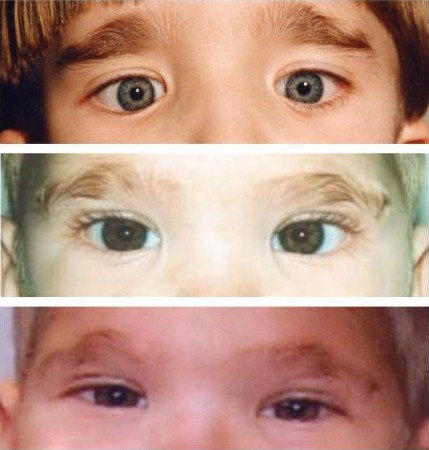
Main common features of the patients with Mowat-Wilson Syndrome: large eyebrows, medially flaring and sparse in the middle part.

In infancy, there are excess nuchal skin, a rounded skull shape, a sparse fine hair and a puffy anterior neck; the face is square shaped with high forehead, frontal bossing, hypertelorism, strabismus, epicanthus, deep set but large eyes, a broad nasal bridge, saddle nose with prominent rounded nasal tip, prominent columella, open mouth, with M-shaped upper lip, frequent smiling, and a prominent but narrow and triangular pointed chin. Additional suggestive facial features include telecanthus, a full or everted lower lip and posteriorly rotated ears. The more consistent and easily recognisable features are the eyebrows, which are large, medially flaring and sparse in the middle part and the ear lobes, which are very typical (Figures 4, 5). They are large and uplifted with a central depression and have been described as being like "orecchiette pasta" or like "red blood corpuscles" in shape.

The facial phenotype of MWS is quite distinct and changes with age: in older children the eyebrows become heavier, broad and horizontal, with an increased wide middle separation and medial sparseness [10,19,22]. The nasal tip lengthens and becomes more depressed, the columella is more prominent, leading to the appearance of a short philtrum, the nasal profile becomes convex, the face tends to elongate and the jaw is more pronounced.

In adolescents, the nasal tip overhangs the philtrum, the face becomes long with prognathism, and a long, pointed or "chisel-shaped" chin [10,19,22]. The uplifted ear lobes do not change significantly with time (with the exception of the central depression, which is less obvious in adults) and are an excellent diagnostic clue.

### Growth

At birth, growth parameters are usually in the normal range, including head circumference. The mean birth weight at term is 3370 g (25^th^–50^th^centile), the mean length is 50.9 cm (50^th^–75^th ^centile) and the mean head circumference is 33 cm (3^rd^–10^th ^centile). The cranial circumference at birth is, in general, one centile less than that of weight and length [12]. Microcephaly is sometimes present at birth, but more often develops gradually in infancy and not all children are microcephalic (at or below 2SD below the mean). Microcephaly is a common but not constant feature and was present in 135 out of 166 of the published cases describing this clinical sign (81%) [19,22,24,29-31]. In the other individuals the head circumference was often between the 3^rd ^and the 10^th ^centile.

Most patients are of slender build. Postnatal short stature is usual (34 out of 73 of the published cases with available information: 46%), but several patients have normal stature.

Information regarding pubertal development in subjects with MWS is, at the moment, limited.

### Neurologic findings and behavior

MWS patients have at least moderate but usually severe mental retardation. Hypotonia is frequent in the first years of life; it was present in 45 out of 48 of the published cases (93%) with available information. Developmental milestones such as sitting and walking are very delayed (mean age of sitting without support is 20 months, and mean age of walking is 4 years and 3 months (range: 23 months to eight years), though some remain non-ambulatory [5,9-12,18,19]. Some patients were noted to have a wide-based or ataxic-like gait, sometimes they held their arms up and flexed at the elbow, reminiscent of individuals with Angelman syndrome [9,19]. Fine motor skills are delayed. The oldest individuals (aged over 20 years) are able to drink from a cup, but need assistance with dressing and activities of daily living [19].

Speech is typically limited to a few words, with onset at around 5–6 years. Some patients do not speak at all. However, reports in the genetic literature, along with anecdotal reports from families and educators, suggest that many patients have receptive language skills and communicate successfully using alternative methods, like sign language [9,10,19].

Most subjects have a happy demeanour with frequent smiling and a happy, affectionate, and sociable personality [9,11,12,19]. Some patients have stereotypes with repeated movements of hands and head, other children are fascinated by turning the pages of books and magazines [19].

Seizures or abnormal electroencephalogram (EEG) are described in 102 out of 139 of the published cases (73%) [19,22,24,29-31], although no particular seizure type is characteristic of the condition. The seizures are variable in nature, from absences to generalized seizures and myoclonic seizures [9,19]. Onset of seizures usually occurs in the second year of life, but seizures may begin in the neonatal period, in infancy or in late childhood, or at over ten years of age [9,10,19]. In some cases, the seizures are resistant to treatment in childhood, but appear to be more easily managed in adolescents and adults [9].

Brain anomalies reported so far include hypoplasia or agenesis of corpus callosum, which was present in 67 out of 155 of the published cases (43%) [19,22,24,29-31], ventriculomegaly [19], cortical atrophy [6,20,25], pachygyria and cerebellar hypoplasia [30,31], poor hippocampal formation [13] and frontotemporal hypoplasia with temporal dysplasia [3,9]. These findings may be under-represented because not all published cases underwent cranial imaging.

### Hirschsprung disease

Hirschsprung disease was present in 97 out of 170 of the published cases (57%) [19,22,24,29,31].

Many patients without known HSCR have been noted to have severe constipation, not investigated by rectal biopsy. Constipation was in fact present in 19 out of 73 of the published cases without HSCR (26%) [19,22,29]. A milder form of HSCR might not be diagnosed in infancy.

The data on the length of the aganglionic segment is incomplete in published cases, but short and long segments are both reported in males and females. Patients with *ZEB2 *deletion, but not those with mutations, tend to develop aganglionosis affecting longer segments [32]. This variety in the severity of HSCR in MWS may be caused by both variations in *ZEB2 *abnormalities and epigenetic factors [32].

It is probable that MWS is under-diagnosed in patients without HSCR and it has been well documented that this feature is not always present [4,5,7-12,19,22,24,25]. In the largest series of 57 cases, the frequency of HSCR was 46%, suggesting that with increasing clinical experience the diagnosis can be made with and without HSCR [22].

### Other oropharyngeal and gastrointestinal findings

Other gastrointestinal anomalies, such as pyloric stenosis (one in case with a family history of pyloric stenosis) have been reported in eight patients [2,7,9,10,19,22,25]. A highly arched palate is often present, possibly secondary to hypotonia [9]. Submucous cleft, cleft soft palate, cleft hard palate, and bilateral cleft lip and palate have been described by some authors [1,3,9,10,20,22,25]. A patient with velopharyngeal insufficiency with laryngomalacia, glossoptosis and micrognathia, as opposed to a prominent chin has been described [19]. This patient had a large deletion encompassing the *ZEB2 *gene, and therefore, it is unclear whether these additional findings may be related to haploinsufficiency of genes surrounding the *ZEB2 *locus [19].

### Congenital heart disease

Congenital heart disease was demonstrated in 87 out of 167 (52%) of the published cases [19,22,24,29-31].

The wide spectrum of heart defects observed include patent ductus arteriosus (16 patients), pulmonary stenosis (12 patients) and ventricular septal defect (12 patients), which are the most common, atrial septal defect (8 patients), pulmonary artery sling (6 patients), Tetralogy of Fallot (5 patients), pulmonary atresia (1 patient), peripheral pulmonary artery stenosis (1 patient), missing pulmonary artery (1 patient), aortic coarctation (4 patients), bicuspid aortic valve (1 patient), interrupted aortic arch (1 patient), mitral prolapse (1 patient) and aortic valve stenosis (1 patient) [1,5,7-14,17-19,22,25].

Structural heart defects are variable, but seem to frequently involve the pulmonary arteries and/or valves.

Five patients have been reported with a rare, unusual congenital heart disease, pulmonary artery sling, two of whom also had tracheal stenosis/hypoplasia [18,19,25]. Another patient had tracheal stenosis with aortic valvular stenosis [22]. In 2007, Dastot-Le Moal suggested that pulmonary artery sling with or without tracheal stenosis may be a particular association of MWS and should prompt the clinician to consider this diagnosis [22].

### Genitourinary

Urogenital/renal anomalies were demonstrated in 81 out of 156 of the published cases (51%) [19,22,24,29-31].

In 63 out of 100 males with MWS for whom this information was available, 58 had genital anomalies, and hypospadias was present in 33/63 patients (52%). This frequency is high for a single anomaly within a multiple congenital anomalies/mental retardation syndrome, where clinical signs can be variably present. From the analysis of EUROCAT data, hypospadias affects about 3 per 1000 male births [33]. Cryptorchidism was described in 36% of patients (23/63), bifid scrotum and webbed penis were identified in 3 out of 63 cases, and micro-phallus in 1 patient. Eight out of 63 males with genitalia anomalies, have both hypospadias and cryptorchidism [5,7,9,12,16-20,24,25,32]. Only one female with MWS had genital anomalies, consisting of a vaginal septum [25].

Other genitourinary anomalies in children with MWS included duplex kidney (1 patient) [1], pelvic kidney (1 patient) [9], vesicoureteric reflux (10 patients) [7,9,10,13,19,22,24] and hydronephrosis (8 patients) [7,10,12,13,18,19].

### Musculoskeletal

Musculoskeletal anomalies occur in many patients. Most individuals are of slender build. In childhood they are described as having slender and tapering fingers, sometimes with prominent fingertip pads, with prominence of the interphalangeal joints developing in later childhood, adolescence and adulthood [9,12,19,34]. The feet have been described as having pes planus, mild calcaneovalgus deformity and long toes [9,12,19,34]. The following features have been reported in at least one affected subject: wormian bones, pectus excavatum, pectus carinatum, superior pectus carinatum/inferior pectus excavatum, scoliosis, genu valgus, recurrent non-traumatic patellar dislocation, mild contractures of the hip, elbows and knees, ulnar deviation of the hands, radial deviation of the thumbs, short broad thumbs, proximally placed thumbs, adducted thumbs (positional), 1^st^–2^nd ^finger syndactyly, distal phalanges of fingers hypoplasia, camptodactyly, delayed bone age, broad halluces, long halluces, hallux valgus, unilateral duplication of the hallux, hypoplasia of halluces, hypertrophic first ray of the feet, middle and distal phalanges hypoplasia of toes, brachytelephalangia, overriding of the 2^nd ^toe over the 1^st ^and 3^rd ^toes [1-3,6,9-11,16,19,20,22,34].

### Eye defects

Eye structural anomalies have only recently been reported in association with MWS and were described in 7 out of 170 of the published cases (4,1%) [18,22]; three had microphthalmia (one of whom with in addition iris coloboma and cataract), three h ad iris/retinal/optic disc colobomas, and one Axenfeld anomaly [17-19,22,28,34]. One patient with coloboma and high myopia had a novel *ZEB2 *missense mutation and trisomy 21 [28]. These eye findings are consistent with the expression of the gene in the developing eye [18].

Strabismus, although quite rarely mentioned, was also evident in many photographs and appears to be frequent. Nystagmus due to fixation difficulties is frequently described in infancy, but tends to resolve with age [9,34].

The following features have been reported in at least one affected subject: ptosis, myopia, astigmatism, dark pigment clumps in blue irises, described as irides heterochromia by some authors [9,12,34].

A pediatric ophthalmologic evaluation should therefore be performed in any individual with MWS.

### Hearing function

Recurrent episodes of otitis media have been described in patients with MWS. Hearing loss was not found in patients tested, although children with recurrent or chronic otitis media are at risk of conductive hearing loss [9,19,34]. An audiological evaluation should be performed in all children with language impairment, including MWS patients [19,34].

### Teeth anomalies

There is limited available information regarding the dental characteristics of patients with MWS. The following features have been reported in at least one affected subject: widely spaced teeth, dental crowding, "malpositioned" teeth, delayed tooth eruption [19,34].

### Skin

One patient with MWS has been described as having gradual onset of widespread "raindrop" depigmentation on the trunk [9,10]. Depigmentation was otherwise reported in two other subjects [3,22]. Dermatoglyphic anomalies/deep palmar and plantar creases have been described in at least eight patients [6,12]. Three subjects have been found to have accessory nipples [19]. Preauricular tag was reported in one individual [1].

### Other clinical features

One patient with MWS had asplenia [18]. Another one had autonomic dysregulation (later onset) [3,22]. Repeated vomiting attacks, suggestive of epilepsy, were observed in five cases [25].

## Etiology

MWS is caused by an heterozygous mutation in the *ZEB2 *gene (OMIM# 605802) [35] that was identified by Wakamatsu *et al *and Cacheux *et al *in 2001 [2,3]. Mowat *et al *described the syndrome in 1998 and also identified a locus at chromosome 2q21-q23 [1].

**Figure 6:**
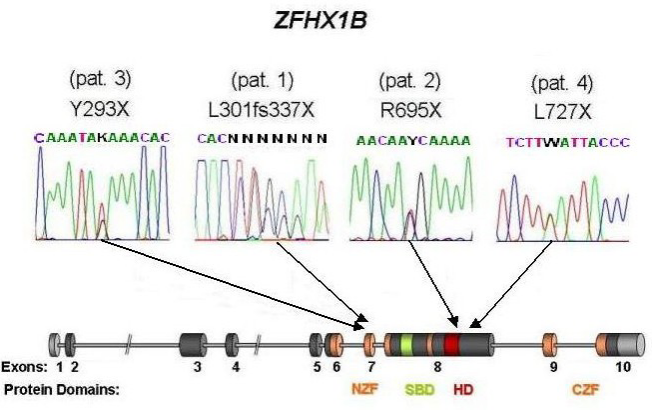
Scheme of *ZEB2 *exons and corresponding protein structure adapted from Zweier *et al *[4], showing the position of the presented mutations. NZF, N-terminal zinc finger cluster; CZF, C-terminal zinc finger cluster; SBD, Smad binding domain; HD, homeodomain like segment. [5, 11, 15, 17, 28].

The *ZEB2 *gene spans approximately 70 Kb, consists of 10 exons and 9 introns (Figure 6), and encodes for SIP1 (Smad interacting protein 1, SMADIP1) [2]. The initiation codon is located in exon 2 and the stop codon is in exon 10. SIP1 is a zinc finger/homeodomain transcriptional repressor and consists of 1214 amino acids [36-39]; *ZEB2 *mRNA is detected in nearly all human tissues [3,8,40,41].

Clinical features suggest that the *ZEB2 *gene is involved in the development of neural-crest derived cells (enteric nervous system, craniofacial mesectoderm), central nervous system, heart septation (patent ductus arteriosus, ventricular and atrial septal defect) and midline development (corpus callosum agenesis, genitourinary anomalies and pyloric stenosis) [5,7,25]. Comparison of the human and mouse homologues of *ZEB2 *at the nucleotide and amino acid levels revealed 93% and 97% similarities, respectively [2,3].

To date, 171 patients have been published [4,9-12,18,19,22,24,29-31]. In all of these patients, heterozygous mutations of the zinc finger E-box-binding homeobox 2 gene (*ZEB2*) were detected. In most cases the mutation produces an absent or truncating protein [9] which loses its function.

The most frequent mutations are: frameshift mutations (small insertions and deletions that lead to truncating frameshift mutations) (41,5%) [2,3,5-8,10,17-19,22,23,25], non-sense mutations (31,6%) [2,5,7,8,10,12,18,22,24,25], and deletions (not detectable by standard cytogenetic method) (19,3%) [2,7,11,18,19,22,24,25,29,31]. Rare mutations have been reported: cytogenetically detectable deletions (1,2%) [1,14], translocation (0,6%) disrupting the disease-gene [3], splice site mutations (2,3%) [10,18,21], missense mutations (1,7%) [20,22,28], complex mutations (combination of deletion and insertion) (1,2%) [22,25] and an inframe mutation (0,6%) [27].

The mutations cover all the coding region [9], and are more frequent in exon 8 (69 cases, 51,1%) [22]. Four recurrent mutations have been observed: 2083C > T (13, exon 8), 2761C > T (5, exon 8) 1027C > T (4, exon 8) and 904C > T (3, exon 7) [8,18,22].

As two patients with non-sense mutations were reported to have the same severe phenotype that those found in patients with large 2q22 deletions, it has been pointed out that MWS is not a contiguous gene syndrome, and that truncating mutations of one allele of the *ZEB2 *gene are sufficient to result in this complex phenotype [12].

Haploinsufficiency is probably the underlying mechanism [7-11,18,22,42].

Few studies on polymorphisms have been carried out. The amino acid altering change p.Pro714Leu, localized in exon 8, was found in one patient (in addition to a non-sense mutation) and in his healthy father, but was not detected in 96 normal controls [18].

The silent alteration p.Ile163Ile was identified both in one patient and in his father [18]. The small number of polymorphisms indicates that *ZEB2 *sequence is under strong evolutionary constraint and even small variations compromise the protein function [18]. A recent study on polymorphisms by Dastot *et al*, 2007, in 180 children (120 unaffected and 60 MWS patients) showed 20% polymorphism in exon 7 (transition T4C in intron 7 (c.917-21T4C) in both groups. The authors identified an amino acid change in exon 10 (amino acid change p.Glu1094Lys) in a typical patient who also had a total deletion of *ZEB2 *on the other chromosome. As this alteration was also identified in the healthy mother, it was probably a polymorphism [22].

In two cases of typical MWS, the mutation was not identified [10]. It could be due to the presence of mutations not detectable by mutational screening, or it could also indicate genetic heterogeneity for this clinical syndrome [10].

## Genotype-phenotype correlation

The studies on genotype-phenotype analysis show that in most cases the phenotype was similar in patients with deletions and in those with truncating mutations [11,12,18]. Patients with large deletions may have a more severe phenotype and additional features [11,25,30,31]. Facial gestalt and the psychomotor development delay, particularly of language, are constant. The frequent and severe malformations (HSCR, congenital heart defect, agenesis of the corpus callosum) and seizures may lead to suspicion of the diagnosis, but their presence is variable [5,9,12,17,18,22].

The comparison between clinical data concerning the associated malformations of one of the patients with a R695X mutation and those of the other eight patients with the same mutation, demonstrated the phenotypic variability of a single mutation in MWS [4,12]. This variability is remarkable not only in the same mutation but also in the same family. In fact, two siblings (sister and brother) with characteristic face, HSCR and agenesis of the corpus callosum were discordant for congenital heart disease and ocular coloboma [17]. On the other hand, the phenotypic expression in two affected sisters was very similar for a/hypoplasia of corpus callosum, HSCR, congenital heart defect, seizures and microcephaly [18]. These features might be controlled by common familial genetic modifiers [18].

Few data are available about the parental origin of deletions. The origin of the deleted chromosome is paternal in 17 out of 19 patients examined so far [11,22,25,42]. The investigation of four patients showed that agenesis of corpus callosum (present in two patients and absent in two others) and seizures (present in one patient and absent in three others) showed no correlation with paternal origin of the deletion [11].

HSCR, when present, is a strong cross reference marker, even in the neonatal period, but it is not constant. It is noteworthy that at first the patients were selected among those with syndromic HSCR. As the number of described patients with *ZEB2 *mutations rises, the percentage of patients with HSCR decreases: 70% of 30 patients [11], 62% of 45 [9], 63.8% of 47 [12], 62.8% of 70 [4], 62,6% of 97 [18], 57.2% of 159 [22] and 57% of 170. Therefore, there was a bias of ascertainment.

Also the male preponderance of HSCR in general populations (4:1) [43] can cause a bias of ascertainment and explain the male excess in MWS [9]. In fact, the male/female ratio decreases from 2.13/1 out of 47 patients (M/F 32/15) [12] to 1.92/1 out of 70 patients (M/F 46/24) [4], to 1.49/1 out of 97 patients (M/F 58/39) (personal data), to 1.37/1 out of 159 patients (M/F 92/67) [22] and today 1.42/1 out of 170 patients (M/F 100/70).

The manifestation of HSCR is not influenced by deletion size [11]. Moreover, *Zfhx1b *knockout mice do not exhibit HSCR [44], therefore a non-allelic modifier might contribute to the manifestation of HSCR [11].

Unusual mutations can lead to an atypical phenotype. The first person reported was a 48-year-old woman with severe constipation and mild mental retardation in the absence of specific facial anomalies, seizures, and other malformations caused by non-truncating mutations with a 3 bp in frame deletion [27].

Three patients with *ZEB2 *missense mutations showed a clinical severity as variable as expected. The first was a boy with Down syndrome and typical facial features of trisomy 21, HSCR (rarer in Down syndrome than in MWS), myopia, and ocular coloboma affecting iris and retina [28]. The child showed some dysmorphism compatible with MWS but not the facial gestalt. The importance of ocular anomalies that differs and are more severe than those observed in Down syndrome is underlined.

Another boy with a missense mutation [20] showed HSCR, corpus callosum hypoplasia, epilepsy and severe mental retardation, but also other anomalies such as cleft lip and palate, brachytelephalangy, and broad thumbs and halluces. Facial phenotype was similar to MWS but differed by the presence of bilateral cleft lip and palate, and eyebrows that were not typical of MWS.

The third missense mutation was found in a young child with typical MWS including HSCR who died at age of 3 years [22].

An exceptionally mild phenotype, caused by a novel and unusual splice site mutation in the 5'UTR, was reported by Zweier *et al *(2006) in a 5-year-old child. He showed a mild motor and speech delay but, by age 5 years, he spoke in full sentences. The phenotype was not typical but facial features resembled the facial gestalt of MWS. Clinical features were mild in comparison with these associated with truncating mutations, no malformations or seizures were present. The mild phenotype could be due to the conservation of all known functional domains of the protein: in fact, this mutation only results in loss of exon 2 [21]. The authors suggested that exon 2 might contain important determinants of the facial phenotype in MWS [21]. Three other splice site mutations [10,18] have been identified in patients with typical phenotypes.

The confirmation of the diagnosis based on the presence of a mutation, deletion or translocation in the *ZEB2 *gene will allow the knowledge on genotype-phenotype correlation to be increased.

## Diagnostic methods

Diagnosis may be suspected on the basis of the clinical phenotype in typical patients. Facial gestalt is particularly important. Serious malformations (HSCR, heart disease, agenesis of corpus callosum) are common even if not always present. Seizures are frequent and psychomotor delay, particularly serious in spoken language, is constant. A small number of patients with rare mutations (inframe, missense and splice site mutations) may show an atypical clinical picture (until now, 2.4% of the patients: 4/169). In all cases, patient should undergo molecular analysis of the *ZEB2 *gene.

A cytogenetic analysis should be carried out to exclude large deletions or translocations. FISH analysis enables submicroscopic deletions to be detected. Sequencing of the complete coding sequence of *ZEB2 *identifies the mutations [5,9,22]. The semi-quantitative fluorescent multiplex polymerase chain reaction (PCR) assay allows detection of other rearrangements that escaped conventional methods [22]. Sequence analysis detects mutations in approximately 79% of affected individuals, FISH analysis detects deletions in 13%, chromosomal rearrangements cause MWS in 2% and an additional 6% have an intermediate-sized deletion detected by quantitative PCR or multiplex ligation-dependent probe amplification (MLPA) [34].

In a patient with multiple congenital anomalies and an apparently balanced translocation involving chromosomes 2, 3 and 5, 1 Mb resolution array-CGH detected a cryptic deletion of about 6 Mb of chromosome 2 including the *ZEB2 *gene [29]. The patient, a six-month-old girl, had dysmorphic features typical of MWS, microcephaly and severe psychomotor retardation.

## Differential diagnosis

The facial features of patients with MWS are quite characteristic, but the presence of HSCR, epilepsy and mental retardation may initially suggest Goldberg-Shprintzen syndrome (GOSHS) [5]. The patients with GOSHS (actually MIM 609460) share clinical features such as HSCR, epilepsy and mental retardation, but have a different facial gestalt (high nasal bridge, synophrys, long curled eyelashes, palpebral ptosis, and cleft palate are commonly observed) [45,46]. The differential diagnosis can be carried out on the basis of facial phenotype and confirmed by mutational analysis of the *ZEB2 *gene. A patient with short segment HSCR, microcephaly, mental retardation, and distinct facial appearance in the absence of *ZEB2 *intragenic mutations was diagnosed as GOSHS [30]. Further molecular studies showed a deletion of the 2q22-q23 region encompassing the *ZEB2 *gene [31].

In five patients with GOSHS in a consanguineous Moroccan family, Brooks *et al *identified a homozygous mutation in *K1AA1279 *at 10q22.1 [47]. Today, it is possible to carry out molecular analyses for both syndromes.

The differential diagnosis is important for genetic counseling, since GOSHS is autosomal recessive, whereas MWS is a sporadic condition.

*ZEB2 *mutation analysis may be also considered in patients with syndromic [43] and apparently non-syndromic HSCR disease and no mutation in other related genes such as *RET *or *EDNRB *[20,48,49].

Individuals with MWS have often been described as having a wide-based or ataxic-like gait, and a smiling, happy and sociable personality. This, combined with absent speech, microcephaly and seizures, has led some individuals to be given a presumptive diagnosis of Angelman syndrome [19]. However, the facial features of MWS, in addition to the other typical congenital anomalies, distinguish these two conditions.

In patients with hypospadias and mental retardation it is necessary to take into account the differential diagnosis with Smith-Lemli-Opitz syndrome, Opitz G/BBB syndrome and X-linked mental retardation-alpha thalassemia syndrome. However, the gestalt of MWS is different.

## Genetic counseling

The majority of patients with MWS so far reported are sporadic cases; therefore the recurrence risk is low. Nevertheless, cases of sibling recurrence have been observed.

Two sisters with MWS born to healthy parents have been reported. Mutation analysis of both parents revealed a low paternal mosaicism. The father had short stature (150 cm, Vietnamese origin), somewhat uplifted earlobes and a pointed nasal tip [18].

Another recurrence was found in a brother and a sister with clinical features of MWS and the same truncating mutation in exon 8. The parents were phenotypically normal, without mutation in the *ZEB2 *gene. Thus, the most likely explanation is germ-line mosaicism [17].

Recently, another family with three affected sibs has been reported [[34] by D. Mowat, personal communication]. Therefore, the recurrence risk, on the basis of the current data, is 3/170 families (1.76%) [17,18,34].

In the case of a balanced familial translocation, the risk is higher. Karyotype and gene analysis should be offered to the parents with an affected child and, in these cases, prenatal diagnosis is possible.

## Antenatal diagnosis

There are few data concerning specific prenatal markers suitable during pregnancy. Wilson *et al *(2003) reported increased nuchal translucency in two patients [10].

Since agenesis of the corpus callosum is the only MWS feature that can be detected prenatally, molecular screening of the *ZEB2 *gene in prenatal isolated agenesis of the corpus callosum, has been carried out [50]. No gene mutations were detected in six fetuses. It has been concluded that *ZEB2 *is not the major gene for isolated agenesis of the corpus callosum and that a particular attention to the facial features (dysplastic ears) at ultrasound or fetopathological examination may help the diagnosis of MWS in fetuses with agenesis of the corpus callosum.

Only one case of examinations in pregnancy has been performed in a family with two children affected by MWS [17]. The first child, a female, had HSCR, dysmorphic features and developmental delay. The cerebral magnetic resonance imaging (MRI) showed agenesis of the corpus callosum but the karyotype analysis was normal; thus, diagnosis was not made. On the second pregnancy, a nuchal translucency scan was reported as normal, but both fetal ultrasound and MRI examination showed agenesis of the corpus callosum. Amniocentesis demonstrated a normal male karyotype [17]. MRI examination of the mother showed a normal corpus callosum. It could be supposed that the absence of diagnosis on the first child did not allow a correlation of agenesis of corpus callosum with the recurrence of a syndrome.

Other observations and studies are necessary to increase the knowledge and identify possible prenatal markers.

## Management

There is no specific treatment for MWS, as the neural defect and also other malformations resulting from the mutation occur in the early stage of embryonal development [41].

The frequent presence of serious congenital malformations requires precocious clinical investigation with intervention of neonatologists, pediatricians and several specialists. Congenital heart disease and HSCR requires early surgery at the first days or months of life. Serious constipation also persists in operated patients.

Seizures can begin in the first months of life and require suitable therapies; however, in two of our patients the seizures were fairly quickly resolved. One patient still has seizures at the age of 9 years, while the other, at the age of six, had never suffered them.

Genitourinary anomalies such as hypospadias, cryptorchidism, bifid scrotum, vesicoureteral reflux and hydronephrosis may be present in the first years of life and may require surgery.

Eye problems are frequent and require a specialized help.

Deafness has not been reported. Nevertheless, speech retardation requires audiometric examination of all MWS children.

All advised vaccinations are recommended.

A periodic follow-up for the different clinical problems should be carried out regularly.

Psychomotor development is retarded in all patients, therefore rehabilitation including physical therapy, psychomotor and speech therapy should be started as soon as possible.

## Prognosis

There are few data about survival of the patients affected by MWS. We are aware of the death of three patients. One patient with a large deletion and seizures in the neonatal period [11], and a patient with aortic valvular stenosis [22] both died in the first month of life. The third patient, having a missense mutation, died at 3 years of age [22].

As far as we know, the oldest patient reported so far is 30-years-old [25].

Early molecular diagnosis is feasible today and is of great importance in order to start the therapeutic and rehabilitation treatment as soon as possible.

The diagnosis of MWS is important also for the family, regardless of the prognosis. In fact, the diagnosis permits caregivers to give information and psychological support, and relieves the families from a profound sensation of solitude.

The increase of knowledge on the syndrome will support the educational and rehabilitation aid by parents and caregivers, and help the achievement of improvements in the psychomotor development and the autonomy of the MWS patients.

The support resulting from Family Associations is also important.
